# Pleiotropy of positive selection in ancient ACE2 suggests an alternative hypothesis for bat-specific adaptations to host coronaviruses

**DOI:** 10.1073/pnas.2321619121

**Published:** 2024-06-04

**Authors:** Yuan-Ting Guo, Ji-Bin Jiang, Guan-Rong Qiao, Rong-Hua Luo, Xin Zhou, Rong Hua, Chang-Bo Zheng, Zhen Liu

**Affiliations:** ^a^College of Life Sciences, Northwest University, Xi’an 710127, China; ^b^State Key Laboratory of Genetic Resources and Evolution, Kunming Institute of Zoology, Chinese Academy of Sciences, Kunming 650201, China; ^c^University of Chinese Academy of Sciences, Beijing 100049, China; ^d^School of Pharmaceutical Science and Yunnan Key Laboratory of Pharmacology for Natural Products, Kunming Medical University, Kunming 650500, China; ^e^Key Laboratory of Animal Models and Human Disease Mechanisms of the Chinese Academy of Sciences, Kunming Institute of Zoology, Chinese Academy of Sciences, Kunming 650201, China; ^f^Yunnan Key Laboratory of Biodiversity Information, Kunming 650201, China; ^g^Key Laboratory of Genetic Evolution and Animal Models, Kunming Institute of Zoology, Chinese Academy of Sciences, Kunming 650201, China

**Keywords:** bat, adaptive evolution, pleiotropy

## Abstract

Angiotensin-convertingenzyme 2 (ACE2) has dual functions, regulating cardiovascular physiology and serving as the receptor for coronaviruses. Bats, the only true flying mammals and natural viral reservoirs, have evolved positive alterations in traits related to both functions of ACE2. This suggests significant evolutionary changes in ACE2 during bat evolution. To test this hypothesis, we examine the selection pressure in ACE2 along the ancestral branch of all bats (AncBat-ACE2), where powered flight and bat-coronavirus coevolution occurred, and detect a positive selection signature. To assess the functional effects of positive selection, we resurrect AncBat-ACE2 and its mutant (AncBat-ACE2-mut) created by replacing the positively selected sites. Compared to AncBat-ACE2-mut, AncBat-ACE2 exhibits stronger enzymatic activity, enhances mice’s performance in exercise fatigue, and shows lower affinity to severe acute respiratory syndrome coronavirus 2 (SARS-CoV-2). Our findings indicate the functional pleiotropy of positive selection in the ancient ACE2 of bats, providing an alternative hypothesis for the evolutionary origin of bats’ defense against coronaviruses.

The coronavirus disease 2019 (COVID-19) pandemic has highlighted the role of angiotensin-converting enzyme 2 (ACE2) as the receptor for SARS-CoV-2, the causative coronavirus (1). However, it is of note that ACE2’s original function is to regulate cardiovascular physiological processes. Deficiency in ACE2 is associated with severe pathological conditions such as dilated cardiomyopathy and cardiac dysfunction (2). Interestingly, there appears to be a close correlation between the two functions of ACE2. For example, both hamsters infected with SARS-CoV-2 and patients with severe COVID-19 commonly exhibit cardiac injuries characterized by functional and structural abnormalities, including arrhythmias, myocardial fibrosis, and heart failure (3).

Bats, which are the only group of mammals capable of true powered flight and serve as the unique natural viral reservoirs, are believed to have evolved positive alterations in traits related to the two functions of ACE2. On the one hand, although bats have been suggested as natural hosts for several coronaviruses that cause infectious diseases, they show minimal or no symptomatic signs of diseases (4, 5). Importantly, ACE2 has been found to undergo episodic selection in residues that interact with coronaviruses among bats (6). On the other hand, the origin and evolution of powered flight have critically impacted the morphology and physiological function of bat hearts, resulting in larger hearts, higher heart rates, and higher metabolic rates (7, 8). These findings suggest that ACE2 in the last common ancestor of bats (AncBat-ACE2) may have experienced remarkable genetic changes driven by selection, potentially influencing its two functions. Our focus on the ancestral branch of all bats is justified because of the long evolutionary history of bats coexisting with viral pathogens (9, 10) and the origination of flight in the last common ancestor of bats (11).

## Results and Discussion

To test our hypothesis, we used a likelihood method based on a modified branch-site model (12) to estimate the selective pressure on AncBat-ACE2. We compiled a dataset that included high-quality protein-coding sequences of ACE2 from 102 representative mammalian species (Dataset S1). The foreground branch was designated as the ancestral branch of all bats, while the other branches served as background branches. The ω value was estimated to be 58.6, and a total of 10 sites were identified as being under positive selection in ACE2 along the ancestral branch of all bats (Dataset S2). The alternative model showed a significantly higher likelihood than the null model (*P* = 0.0012; two-tailed *χ*^2^ test), suggesting that ACE2 underwent positive selection along the ancestral branch of all bats. Notably, rhinolophid bats have been more frequently identified as hosts for SARS-related viruses compared to other bat clades (13). We thus examined the evolutionary pressure on ACE2 along the branch of rhinolophid bats and did not find a significant signal of positive selection. This suggests that rhinolophid bats may have evolved alternative mechanisms for hosting SARS-related viruses. Together, these findings indicate that ACE2 has undergone positive Darwinian selection along the ancestral branch of all bats, potentially resulting in functional changes in AncBat-ACE2.

**Fig. 1.**
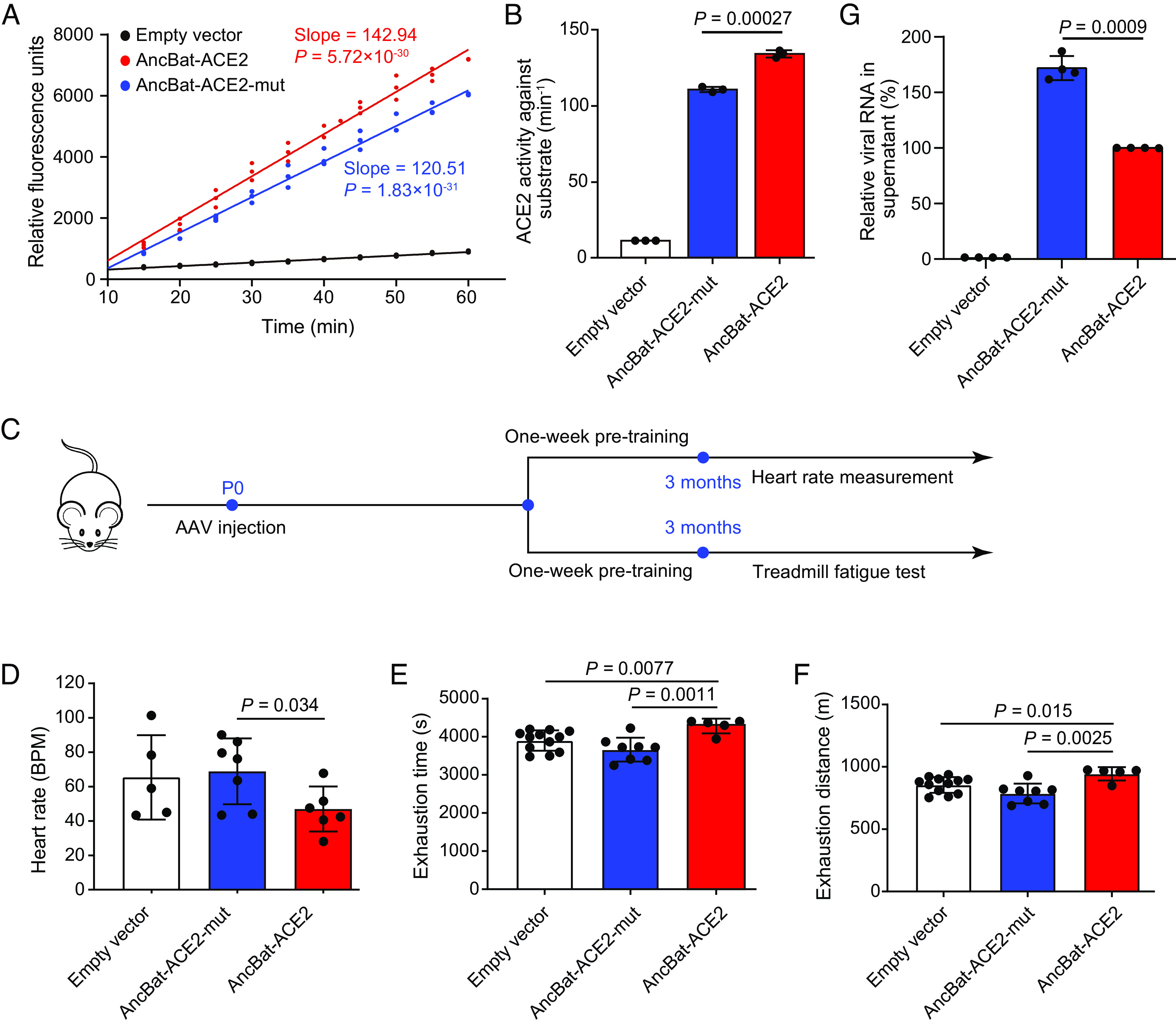
Functional pleiotropy of positive selection in AncBat-ACE2. (*A*) Detection of enzymatic activities of AncBat-ACE2 and AncBat-ACE2-mut. The curves of the relative fluorescence intensity of the catalytic product over time for AncBat-ACE2 and AncBat-ACE2-mut are presented. The slope of the curve represents the enzymatic activity of ACE2. (*B*) The enzymatic activity of AncBat-ACE2 is significantly higher than that mediated by AncBat-ACE2-mut. (*C*) An illustration of behavioral experiments of mice carrying AncBat-ACE2 and AncBat-ACE2-mut. (*D*) The heart rates for mice carrying AncBat-ACE2 and AncBat-ACE2-mut are compared before and after injecting dobutamine hydrochloride. BPM represents beats per minute. (*E*) The exhaustion time of mice carrying AncBat-ACE2 and AncBat-ACE2-mut is compared. (*F*) The exhaustion distance of mice carrying AncBat-ACE2 is significantly greater than that of mice carrying AncBat-ACE2-mut. (*G*) Comparison of the relative amount of SARS-CoV-2 RNA in the supernatant mediated by AncBat-ACE2 and by AncBat-ACE2-mut. The dots indicate the number of biological replicates. The *P* values are from the two-tailed Student’s *t* test.

Among the 10 positively selected sites (PSSs) on the ancestral branch of all bats, one site (H34T) is involved in contacting SARS-CoVs, five sites (D367E, I407V, A412V, I421M, and T445N) are located in the catalytic domain, two sites (A99I and A193G) are in the zinc-containing subdomain, and the remaining two sites (Y521F and S607H) are in the C-terminus-containing subdomain (14). These locations suggest that the positive selection may be related to both enzymatic activity and coronavirus binding of AncBat-ACE2. To assess whether the PSSs influence the enzymatic activity of AncBat-ACE2, we used the maximum likelihood method to infer the protein-coding sequence of AncBat-ACE2. The inference was considered reliable as the mean posterior probabilities for the entire sequence of AncBat-ACE2 exceeded 95%. Subsequently, we generated a corresponding mutant of AncBat-ACE2 by artificially replacing the PSSs in AncBat-ACE2 with those found in outgroups (AncBat-ACE2-mut; Dataset S3). The two protein-coding sequences of AncBat-ACE2 and AncBat-ACE2-mut were synthesized and used in the enzymatic activity assay. We conducted fluorescence intensity measurements generated from catalyzed substrates using equal amounts of proteins from AncBat-ACE2 and AncBat-ACE2-mut (Dataset S4). Upon comparing the results, we observed that the slope of the standard curve from the relative fluorescence units (Fig. 1*A*), representing ACE2 activity against substrate per unit time, was significantly larger for AncBat-ACE2 than for AncBat-ACE2-mut (*P* = 0.00027, two-tailed Student’s *t* test; Fig. 1*B*). This result suggests that positive selection has led to the enhanced enzymatic activity of AncBat-ACE2, as the PSSs were the only sequence differences between AncBat-ACE2 and AncBat-ACE2-mut.

The increased enzymatic activity of AncBat-ACE2 may reflect adaptations to flight in bats, such as enhanced cardiac function and energy metabolism. To test this hypothesis, we generated mouse models using in vivo gene transfer mediated by adeno-associated virus 9 (AAV9) (Fig. 1*C*). Neonatal mice were treated with intravenous injections of AAV vectors carrying AncBat-ACE2 and AncBat-ACE2-mut at a dose of 1 × 10^11^ virus copies per pup, which was sufficient to transduce cardiac cells, as confirmed by PCR using specific primers after 3 mo of AAV transduction. We then measured heart rates in two groups of mice, one carrying AncBat-ACE2 and the other carrying AncBat-ACE2-mut. While there was no significant difference in resting heart rates, the change in heart rate before and after injecting dobutamine hydrochloride, a commonly used inotropic agent for increasing cardiac output, was significantly lower in mice carrying AncBat-ACE2 compared to mice carrying AncBat-ACE2-mut (*P* = 0.034; two-tailed Student’s *t* test; Fig. 1*D*). This result suggests that mice carrying AncBat-ACE2 may have a stronger capacity for regulating heart rate and cardiac function. To confirm this suggestion, we conducted a treadmill test on the two groups of mice and measured the time and distance they were able to run until exhaustion. On average, mice carrying AncBat-ACE2 ran for 4,117.4 ± 327 (mean ± SD) seconds and covered a distance of 944.7 ± 54 m before exhaustion, which were significantly larger than those from mice carrying AncBat-ACE2-mut (*P* = 0.0011, *P* = 0.0025; two-tailed Student’s *t* tests; Fig. 1 *E* and *F*). Overall, our findings indicate that positive selection leads to stronger enzymatic activity of AncBat-ACE2 and, more importantly, improves cardiac function and energy metabolism in vivo.

Given the suggestion of functional pleiotropy reflected by the locations of the PSSs, we investigated whether positive selection influence the change in infection efficiency of SARS-CoV-2 mediated by AncBat-ACE2. We first conducted protein docking between AncBat-ACE2 and the receptor binding domain (RBD) of SARS-CoV-2, as well as between AncBat-ACE2-mut and the RBD of SARS-CoV-2, using the ZDOCK server (15). Both the binding energy and the dissociation constant suggest a higher binding affinity of AncBat-ACE2-mut with the RBD of SARS-CoV-2 compared to AncBat-ACE2 (Dataset S5). This result was highly consistent with that between the RBD of SARS-CoV-2 and the AncBat-ACE2-mut-2, which was created by artificially replacing the five PSSs not in the catalytic domain of ACE2.

To confirm this result, we, respectively, transfected AncBat-ACE2 and AncBat-ACE2-mut in the A549 cell line and successfully expressed them at comparable levels. The relative quantity of SARS-CoV-2 RNA in the supernatant, mediated by AncBat-ACE2, was significantly lower than that mediated by AncBat-ACE2-mut (*P* = 0.0009; two-tailed Student’s *t* test; Fig. 1*G*). This suggests that the PSSs in AncBat-ACE2 are closely associated with the change in infection efficiency of SARS-CoV-2, further supporting the pleiotropy of positive selection of ACE2 on the ancestral branch of all bats.

In summary, our study identified adaptive selection occurring in ACE2 of the last common ancestor of bats. Through a combination of gene functional experiments and animal behavioral experiments, we demonstrated the functional pleiotropy of positive selection in the ancient ACE2 of all bats. It is challenging to definitively determine which PSSs are more crucial for enhanced enzymatic activity or weaker binding to coronaviruses of AncBat-ACE2. Assuming that the PSSs contribute individually and specifically to these two functional aspects of AncBat-ACE2, the consistently higher binding affinity between AncBat- ACE2-mut and AncBat-ACE2-mut-2 suggests that the five PSSs in the catalytic domain of ACE2 may enhance the enzymatic activity of AncBat-ACE2 and improve the performance of mice in treadmill fatigue tests. This suggestion will require further experiments to be tested in the future. Our findings suggest a potential link between host defenses against coronaviruses and improved cardiac function following the origin of flight in bats, providing an alternative hypothesis for the evolutionary origin of bats’ defense against coronaviruses.

## Materials and Methods

Based on the ACE2 protein-coding sequences of 102 representative mammalian species, we examined the evolutionary pressures on the ancestral branch of all bats and reconstructed the ACE2 protein-coding sequence of the last common ancestor of all bats (AncBat-ACE2) using the branch-site likelihood method. We synthesized and cloned the protein-coding sequences of AncBat-ACE2 and its mutant (AncBat-ACE2-mut) into expression vectors to perform their enzymatic activity assay and viral infection experiments. After delivering AncBat-ACE2 and AncBat-ACE2-mut to mouse hearts using AAV, we measured the heart rate of the mice and conducted treadmill fatigue tests. Details are available in *SI Appendix*.

## Supplementary Material

Appendix 01 (PDF)

Dataset S01 (XLSX)

Dataset S02 (XLSX)

Dataset S03 (XLSX)

Dataset S04 (XLSX)

Dataset S05 (XLSX)

Dataset S06 (XLSX)

## Data Availability

All study data are included in the article and/or supporting information.
