# Supplementary material for: Pleiotropy of positive selection in ancient ACE2 suggests an alternative hypothesis for bat-specific adaptations to host coronaviruses
Source: Proc Natl Acad Sci U S A. 2024 Jun 4;121(24):e2321619121. doi: 10.1073/pnas.2321619121 (PMC11181079; doi:10.1073/pnas.2321619121)
Supplement: Supplementary file 1 — Appendix 01 (PDF) [file pnas.2321619121.sapp.pdf]

Supplementary Information

for

Pleiotropy of positive selection in ancient ACE2 suggests an alternative hypothesis for bat-specific adaptations to host coronaviruses

Yuan-Ting Guo, Ji-Bin Jiang, Guan-Rong Qiao, Rong-Hua Luo, Xin Zhou, Rong Hua, Chang-Bo Zheng*, and Zhen Liu*

*Correspondence to:

Chang-Bo Zheng

Email: zhengchangbo@kmmu.edu.cn

&

Zhen Liu

Email: zhenliu@mail.kiz.ac.cn

This PDF file includes:

Materials and methods

SI References

Other supplementary materials for this manuscript include the following:

Dataset S1

Dataset S2

Dataset S3

Dataset S4

Dataset S5

Dataset S6

Materials and Methods

Protein-coding sequences of ACE2 and evolutionary analyses

To reduce bias caused by an unequal number of closely related species in subsequent evolutionary analyses, we selected one representative species from each of the 102 mammalian genera that had intact and high-quality ACE2 protein-coding sequences (**Dataset S1**). We aligned these ACE2 protein-coding sequences using the MACSE2 program (1) with default parameters and filtered the alignment using HmmCleaner (2) to reduce the potential false positives in the alignment. Based on the phylogeny of these species obtained from the TimeTree database (3), we examined the evolutionary pressures on the ancestral branch of all bats using the likelihood method implemented in PAML4 (4) under the branch-site model with no molecular clock and an unrooted tree (see details in **Dataset S6**). This method involved comparing the likelihood ratios between two models: the alternative model (Model A) and the null model (Model A0). Model A categorized sites into four classes based on the ω value, which is the ratio of nonsynonymous (d_N) to synonymous (d_S) nucleotide substitution rates. Site class 0 represents conserved codons throughout the tree with an estimated ω value between 0 and 1 ($0 < \omega_0 < 1$). Site class 1 includes neutral codons throughout the tree with an estimated ω value of 1 ($\omega_1 = 1$). Site class 2a consists of codons that are conserved on background branches compared to foreground branches with an estimated ω value between 0 and 1 ($0 < \omega_{2a} < 1$). These codons are assumed to be under positive selection ($\omega_2 > 1$) on the foreground branches. Site class 2b comprises codons assumed to be neutral on the background branches but under positive selection on the foreground branch with an estimated ω value greater than 1 ($\omega_2 > 1$). In the corresponding Model A0, the value of ω_2 is fixed at 1. A likelihood ratio test is conducted to compare the two models. If the likelihood of Model A is significantly different from that of Model A0, it is interpreted as evidence of positive selection on the foreground branches. Meanwhile, the positively selected sites are inferred using Bayes Empirical Bayes prediction (5). To ensure the reliability of our evolutionary analyses, we performed the PAML analysis five times, each time assigning different estimated parameters (kappa = 1, 2, 3, 4, or 5) and found that the results from these analyses were highly consistent. The ACE2 protein-coding sequence of the last common ancestor of all bats (AncBat-ACE2) was reconstructed using the maximum likelihood method implemented in PAML4. The inference was considered reliable as the mean posterior probabilities for the entire sequence exceeded 95%.

ACE2 enzymatic activity assay

The intact protein-coding sequences of AncBat-ACE2 and its mutant (AncBat-ACE2-mut), which was created by artificially replacing the positively selected sites in AncBat-ACE2 with their corresponding sites in outgroups, were synthesized (Sangon Biotech, China). The synthesized sequences of AncBat-ACE2 and AncBat-ACE2-mut were cloned into the PHBLV-CMV-3FLAG-GFP empty vector (Hanbio Biotechnology, Shanghai), respectively. HEK 293T cells were seeded at a density of 2.6×10^6 cells in a 6-cm dish and allowed to adhere overnight. The cells in each dish

were then transfected with 6 μg of plasmids using the standard protocol for Lipofectamine 3000 (Thermo Fisher Scientific). The cell culture medium was refreshed after 12 hours. After 48 hours of transfection, fluorescent cells were collected to assess the enzymatic activity of AncBat-ACE2 and AncBat-ACE2-mut using the ACE2 Activity Fluorometric Assay Kit (Beyotime, Shanghai). Briefly, the cells were lysed using the Lysis Buffer in the kit and the total protein concentrations were determined using the BCA Protein Assay Kit (Pierce, Rockford). For the Western Blotting analysis of ACE2, 20 μg total protein from each sample was used. The ACE2 protein levels were assessed by measuring the intensity of the color rendered by the 3FLAG tag antibody using ImageJ software. To ensure equal protein amounts for AncBat-ACE2 and AncBat-ACE2-mut, they were diluted to a concentration of 1 μg/μL using the assay buffer in the kit. Then, 10 μL of the ACE2 protein dilution was mixed with 88 μL of assay buffer and reacted with 2 μL of substrate. The relative fluorescence values were measured using a FlexStation 3 plate reader (Molecular Devices) at 5-minute intervals for a total of 60 minutes.

Adeno-associated virus (AAV) delivering AncBat-ACE2 and AncBat-ACE2-mut to mouse heart

We followed a previous study (6) to create the cardiomyocyte-specific gene expression vector pAAV-cTnT. Briefly, the chicken cTnT promoter was cloned into pAAV-MCS (Addgene) by replacing the CMV promotor. The protein-coding sequences of AncBat-ACE2 and AncBat-ACE2-mut were amplified and inserted into the pAAV-cTnT vector, yielding the over-expression vectors of pAAV-cTnT-ACE2. Recombinant vectors, pRC9(Addgene) and pHelper (Cell Biolabs) were purified following the manufacturer's instructions (Qiagen). HEK293T cells (4×10^8) were seeded in a 10-layer cell factory and co-transfected with plasmids using polyethylenimine. After 6 hours, the supernatant was replaced with fresh medium. After 72 hours, the cells were harvested and lysed using a lysis buffer (50 mM Tris-Cl, 150 mM NaCl, 2 mM $MgCl_2$, 0.1% Triton X-100). The cell lysates were treated with Benzonase (Merck) at a concentration of 50 U/ml and incubated at 37°C for 1 hour. Subsequently, the supernatants were filtered using 0.22 μm filters. The AAV9 vectors were then purified using affinity Chromatography (Thermo Fisher) and ion-exchange column chromatography (BIA, 311.5113-2) following the manufacturer's instructions. To further purify the AAV9 vectors, the viral solution was subjected to Q-anion exchange chromatography, which exploits the difference in iso-electric points between the viral capsid and impurities. Finally, the AA9 viral solution was tittered using quantitative polymerase chain reaction (qPCR) targeting the ITR (inverted terminal repeat) region in the AAV-TnT vectors.

C57BL/6J mice were housed in the animal center at the Kunming Institute of Zoology, Chinese Academy of Sciences. All animal experiments were conducted following the guidelines and regulations set by the Institutional Animal Care and Use Committee (IACUC), Kunming Institute of Zoology, Chinese Academy of Sciences. The obtained AAV9 vectors were injected into neonatal C57BL/6J mice using a previously described procedure (7). Briefly, p0 pups were anesthetized on ice for 30

seconds, and then $1×10^{11}$ virus copies of the AAV9 viral solution were slowly injected through the superficial temporal vein. After 3 months, PCR amplification was performed using the specific primers designed for bat-specific sequences to confirm the expression of AncBat-ACE2 and AncBat-ACE2-mut in the heart of the treated mice. The forward primer used was GAGTCACCTGGCCCTGGGTA, and the reverse primer used was AGTTGGACTGATATGGAA.

Heart rate measurement

We utilized a non-invasive blood pressure detector (MRBP-M01, IITC) to measure the heart rates of the mice carrying AncBat-ACE2 and AncBat-ACE2-mut. Before the measurements, each mouse underwent a minimum of one week of adaptive training to minimize heart rate changes caused by emotional factors such as nervousness towards the instrument. Any mouse that failed to complete the pre-training successfully was excluded from the study. During the heart rate measurements, the heating device was activated to maintain an internal temperature of 37°C. The pulse sleeve was placed on the tail of the mouse, near the 1/3 position of the heart, and the mouse was allowed to adapt to the instrument for 10-20 minutes. The sensor automatically recorded the heart rates of the mice. Three consecutive measurements were performed on each mouse, and the average value was calculated. After completing the initial heart rate measurement, dobutamine hydrochloride was intraperitoneally injected into the mouse. The mouse was then placed back into the instrument and after a 5-minute interval, its heart rate was measured again.

Treadmill fatigue test

Following the protocol described in a previous study (8), we conducted the treadmill fatigue test on two groups of mice carrying AncBat-ACE2 and AncBat-ACE2-mut, respectively. Briefly, the pre-training for the treadmill test was conducted one week before the formal treadmill test. During pre-training, the platform angle was set to 10°, and the electrical stimulation current was set to 1.22 mA to minimize potential harm to the animals. The mouse that failed to complete the pre-training successfully was excluded from the study. Notably, the mice used to measure heart rate changes were unsuitable for the treadmill fatigue tests due to drug injection. Based on the pre-training results, it was determined that a speed parameter of 15 m/min was most suitable, allowing the mice to perform the running task effectively. During the formal treadmill test, the time and distance to exhaustion were recorded. The criterion for exhaustion was defined as the mice being unable to continue running despite receiving continuous electric shots for 5 seconds.

Docking prediction between ACE2 and the receptor-binding domain of SARS-CoV-2

We created a protein sequence of AncBat-ACE2-mut-2 by artificially replacing the five positively selected sites (H34T, A99I, A193G, Y521F, and S607H) in AncBat-ACE2 with the corresponding sites found in outgroups. These five sites are not located in the catalytic domain of ACE2. We used the SWISSMODEL server (https://swissmodel.expasy.org/) to build the three-dimensional (3D) structures of the

N-terminal peptidase domain (residues Ser19 - Asp615) of AncBat-ACE2, AncBat-ACE2-mut, and AncBat-ACE2-mut-2. The quality of these models was validated using ProSAweb (https://prosa.services.came.sbg.ac.at/prosa.php). We obtained the crystal structure of the SARS-CoV-2 spike receptor-binding domain bound with human ACE2 (PDB ID: 6M0j) from the Protein Data Bank (https://doi.org/10.2210/pdb6m0j/pdb). Using the PhyMol software (https://pymol.org/2/), we extracted the 3D structure of the RBD of SARS-CoV-2. To investigate the interaction between the ACE2 proteins (AncBat-ACE2, AncBat-ACE2-mut, and AncBat-ACE2-mut-2) and the RBD of SARS-CoV-2, we utilized the Z-Dock server (9) for protein docking. The binding affinity, the dissociation constant, and the interface area were analyzed using both the PRODIGY server (10) and PDBePISA server (https://www.ebi.ac.uk/pdbe/pisa/).

SARS-CoV-2 infection assay

The intact protein-coding sequences of AncBat-ACE2 and AncBat-ACE2-mut were cloned into the expression vector pcDNA3.1, resulting in C-terminal GFP fusion constructs. The human lung epithelial cell line A549, purchased from ATCC (Lot# CCL-185), was cultured in DMEM (Sangon Biotech, China) supplemented with 10% fetal bovine serum (Thermo Fisher Scientific). The human lung epithelial A549 cells were seeded at a density of 3×10^5 cells per well in a 24-well plate and allowed to adhere overnight. The cells in each well were then transfected with 0.5 μg of plasmids according to the standard protocol for Lipofectamine 3000 (Thermo Fisher Scientific). The cell culture medium was refreshed every 6 hours after transfection. After 24 hours of transfection, the fluorescent cells were transferred to the Biosafety Level 3 (BSL3) laboratory and infected with SARS-CoV-2 (strain 107) at a multiplicity of infection (MOI) of 2.7. The SARS-CoV-2 strain 107 [GBW(E)091089] was obtained from the Guangdong Provincial Center for Disease Control and Prevention (Guangzhou, China). Each infection experiment was performed with four independent replicates. Following one hour of post-infection, the free virus was removed by washing the cells three times with PBS. The cells were then incubated in a DMEM fresh medium containing 3% FBS in a 37℃ incubator with 5% CO_2. After 48 hours, the supernatant of the cells was collected. The viral RNA was extracted using the High pure Viral RNA Kit (Roche), and the SARS-CoV-2 load was determined through quantitative PCR using the One-Step qRT-PCR Kit (Toyobo) with the primers targeting the nucleocapsid protein (N). The primers and probe were as follows: forward (5’-GGGGAACTTCTCCTGCTAGAAT-3’), reverse (5’-CAGACATTTTGCTCTCAAGCTG-3’), and probe (5’-[FAM]TTGCTGCTGCTTGACAGATT[TAMRA]-3’). All infection experiments were conducted at BSL3 of the Kunming Institute of Zoology, Chinese Academy of Sciences.

References

1. V. Ranwez, N. Chantret, F. Delsuc, Aligning protein-coding nucleotide sequences with MACSE. *Methods Mol. Biol.* **2231**, 51-70 (2021).
2. A. Di Franco, R. Poujol, D. Baurain, H. Philippe, Evaluating the usefulness of alignment filtering methods to reduce the impact of errors on evolutionary inferences. *BMC Evol. Biol.* **19**, 21 (2019).
3. S. Kumar et al., TimeTree 5: An expanded resource for species divergence times. *Mol. Biol. Evol.* **39**, msac174 (2022).
4. Z. Yang, PAML 4: phylogenetic analysis by maximum likelihood. *Mol. Biol. Evol.* **24**, 1586-1591 (2007).
5. Z. Yang, W. S. Wong, R. Nielsen, Bayes empirical bayes inference of amino acid sites under positive selection. *Mol. Biol. Evol.* **22**, 1107-1118 (2005).
6. K. M. Prasad, Y. Xu, Z. Yang, S. T. Acton, B. A. French, Robust cardiomyocyte-specific gene expression following systemic injection of AAV: in vivo gene delivery follows a Poisson distribution. *Gene Ther.* **18**, 43-52 (2011).
7. D. J. Gessler, P. W. L. Tai, J. Li, G. Gao, Intravenous infusion of AAV for widespread gene delivery to the nervous system. *Methods Mol. Biol.* **1950**, 143-163 (2019).
8. J. P. Dougherty, D. A. Springer, M. C. Gershengorn, The treadmill fatigue test: A simple, high-throughput assay of fatigue-like behavior for the mouse. *Jove-J. Vis. Exp.* **111**, e54052 (2016).
9. B. G. Pierce, Y. Hourai, Z. Weng, Accelerating protein docking in ZDOCK using an advanced 3D convolution library. *PLoS One* **6**, e24657 (2011).
10. L. C. Xue, J. P. Rodrigues, P. L. Kastritis, A. M. Bonvin, A. Vangone, PRODIGY: a web server for predicting the binding affinity of protein-protein complexes. *Bioinformatics* 32, 3676-3678 (2016).
